# Repeat expansions in *NOP56* are a cause of spinocerebellar ataxia Type 36 in the British population

**DOI:** 10.1093/braincomms/fcad244

**Published:** 2023-09-14

**Authors:** Tanya Lam, Clarissa Rocca, Kristina Ibanez, Anupriya Dalmia, Samuel Tallman, Marios Hadjivassiliou, Anke Hensiek, Andrea Nemeth, Stefano Facchini, Genomics England Research Consortium, J C Ambrose, P Arumugam, R Bevers, M Bleda, F Boardman-Pretty, C R Boustred, H Brittain, M A Brown, M J Caulfield, G C Chan, A Giess, J N Griffin, A Hamblin, S Henderson, T J P Hubbard, R Jackson, L J Jones, D Kasperaviciute, M Kayikci, A Kousathanas, L Lahnstein, A Lakey, S E A Leigh, I U S Leong, F J Lopez, F Maleady-Crowe, M McEntagart, F Minneci, J Mitchell, L Moutsianas, M Mueller, N Murugaesu, A C Need, P O’Donovan, C A Odhams, C Patch, D Perez-Gil, M B Pereira, J Pullinger, T Rahim, A Rendon, T Rogers, K Savage, K Sawant, R H Scott, A Siddiq, A Sieghart, S C Smith, A Sosinsky, A Stuckey, M Tanguy, A L Taylor Tavares, E R A Thomas, S R Thompson, A Tucci, M J Welland, E Williams, K Witkowska, S M Wood, M Zarowiecki, Nicholas Wood, Andrea Cortese, Henry Houlden, Arianna Tucci

**Affiliations:** Department of Clinical Genetics, Great Ormond Street Hospital NHS Trust, London, WC1N 3JH, UK; Clinical Pharmacology, William Harvey Research Institute, School of Medicine and Dentistry, Queen Mary University of London, London, EC1M 6BQ, UK; Department of Neuromuscular Diseases, UCL Queen Square Institute of Neurology, London, WC1N 3BG, UK; Clinical Pharmacology, William Harvey Research Institute, School of Medicine and Dentistry, Queen Mary University of London, London, EC1M 6BQ, UK; Clinical Pharmacology, William Harvey Research Institute, School of Medicine and Dentistry, Queen Mary University of London, London, EC1M 6BQ, UK; Genomics England, London, E14 5AB, UK; Academic Department of Neurosciences and Neuroradiology, Sheffield Teaching Hospitals NHS Trust, Sheffield, S10 2JF, UK; Department of Clinical Neurosciences, Addenbrookes Hospital, Cambridge, CB2 0QQ, UK; Oxford Centre for Genomic Medicine, Oxford University Hospitals National Health Service Foundation Trust, Oxford, OX3 9DU, UK; Department of Neuromuscular Diseases, UCL Queen Square Institute of Neurology, London, WC1N 3BG, UK; Department of Neuromuscular Diseases, UCL Queen Square Institute of Neurology, London, WC1N 3BG, UK; Department of Neuromuscular Diseases, UCL Queen Square Institute of Neurology, London, WC1N 3BG, UK; Department of Brain and Behavioural Sciences, University of Pavia, Pavia, 27100, Italy; Department of Neuromuscular Diseases, UCL Queen Square Institute of Neurology, London, WC1N 3BG, UK; Clinical Pharmacology, William Harvey Research Institute, School of Medicine and Dentistry, Queen Mary University of London, London, EC1M 6BQ, UK

**Keywords:** spinocerebellar ataxia, NOP56, SCA36, repeat expansion, whole-genome sequencing

## Abstract

Spinocerebellar ataxias form a clinically and genetically heterogeneous group of neurodegenerative disorders characterized by progressive cerebellar ataxia. Their prevalence varies among populations and ethnicities. Spinocerebellar ataxia 36 is caused by a GGCCTG repeat expansion in the first intron of the *NOP56* gene and is characterized by late-onset ataxia, sensorineural hearing loss and upper and lower motor neuron signs, including tongue fasciculations. Spinocerebellar ataxia 36 has been described mainly in East Asian and Western European patients and was thought to be absent in the British population. Leveraging novel bioinformatic tools to detect repeat expansions from whole-genome sequencing, we analyse the *NOP56* repeat in 1257 British patients with hereditary ataxia and in 7506 unrelated controls. We identify pathogenic repeat expansions in five families (seven patients), representing the first cohort of White British descent patients with spinocerebellar ataxia 36. Employing *in silico* approaches using whole-genome sequencing data, we found an 87 kb shared haplotype in among the affected individuals from five families around the *NOP56* repeat region, although this block was also shared between several controls, suggesting that the repeat arises on a permissive haplotype. Clinically, the patients presented with slowly progressive cerebellar ataxia with a low rate of hearing loss and variable rates of motor neuron impairment. Our findings show that the *NOP56* expansion causes ataxia in the British population and that spinocerebellar ataxia 36 can be suspected in patients with a late-onset, slowly progressive ataxia, even without the findings of hearing loss and tongue fasciculation.

## Teaser

Lam *et al*. identified five families of British descent affected by spinocerebellar ataxia (SCA) Type 36, which was thought to be absent in the British population. They detected a pathogenic expansion in the *NOP56* gene. The phenotypic presentation of the probands was in keeping with previously reported patients affected by SCA Type 36.

## Graphical Abstract

**Graphical Abstract:**
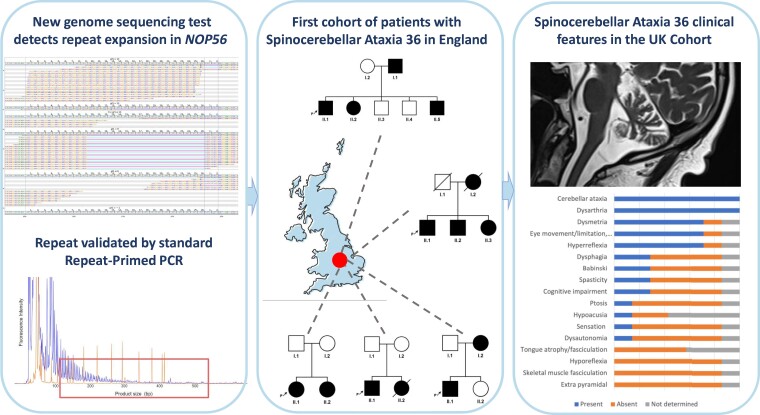

## Introduction

Spinocerebellar ataxias (SCAs) are a group of heterogeneous, neurodegenerative conditions that have the common features of progressive cerebellar ataxia, dysarthria and visual problems.^1^ There is a significant overlap of clinical features between subtypes, although some characteristic features assist in distinguishing between the 49 subtypes that have been classified to date. These hereditary diseases are inherited in an autosomal-dominant pattern, most commonly due not only to repeat expansions but also to point mutations and deletions.^2^ Globally, the prevalence of SCAs is 3 in 100 000; however, prevalence varies largely in different populations.^1,3^

SCA36 is caused by large intronic repeat expansions of a hexanucleotide GGCCTG in the first intron of the *NOP56* gene. The normal alleles contain 3–14 hexanucleotide repeats, while the expanded alleles range from 30 to 2500 repeats (mostly between 650 and 2500 repeats).^4^*NOP56* encodes a ubiquitously expressed core scaffolding protein that stabilizes the box C/D small nucleolar ribonuclear protein complex. The small nucleolar ribonuclear protein complex catalyses the methylation of ribosomal RNAs.^5^ Hypotheses of pathogenesis in SCA36 include haploinsufficiency, gain-of-function of repeat RNA and unconventional translation of repeat peptides. Clinically, SCA36 is characterized by a relatively late-onset cerebellar ataxia, on average in the fifth to sixth decade, with high rates of hearing impairment, which were first described as the most common initial features.^4,6–11^ Accompanying initial features include dysarthria,^4,6,7,12^ dysphagia^6,8,13^ and vertigo and upper and lower motor neuron signs.^4,6^ The progression of disease is generally slow,^4,12^ with the most rapid decline to requiring wheelchair described in 5 years.^9^

Since first being described in Western Japan^7,14^ and given the name ‘Asidan’ after the region’s river, SCA36 was also identified in a large cohort in Costa del Morte, Galicia, Spain, with a common ancestor.^6,15^ Other reports of SCA36 have since been described predominantly in East Asia (mostly Han Chinese and Japan) and Western Europe (Galicia in Spain and neighbouring countries including France) for a total of ∼150 patients from 73 pedigrees (Supplementary Table 1). SCA36 accounted for 1.9% of ataxia cases in the French population and 6.3% in the Galician region of Spain.^4,6^ In East Asia, SCA36 accounted for between 0.6 and 1.6% of SCA.^8,11^ Genetic screening of SCA36 in patients with ataxia in other parts of Europe, including the UK, Greece, Germany and Portugal, failed to identify patients carrying the *NOP56* expansion.^16–18^

Recently developed bioinformatics tools that identify repeat expansions from whole-genome sequencing (WGS) have enabled the discovery of novel repeat expansion disorders in addition to diagnosing patients with known repeat expansion loci.^19–22^

In this study, we combine bioinformatic analysis, together with repeat-primed polymerase chain reaction (RP-PCR) and optical genome mapping, to analyse SCA36 in the British population, including a large cohort of ataxia patients and unrelated controls. We identify five families (seven patients) with the *NOP56* repeat expansion, representing the first report of White British descent patients with SCA36.

## Materials and methods

### Patients

Patients with genetically undiagnosed rare disease and their family members were recruited for the 100 000 Genomes Project by neurologists and clinical geneticists at English hospitals. The consent was obtained from all patients following ethical approval from the National Research Ethics Committee (14/EE/1112).

At recruitment, standardized clinical data were recorded using the Human Phenotyping Ontology, according to disease-specific data model (https://www.genomicsengland.co.uk/?wpdmdl=5500). Following the identification of patients carrying a repeat expansion in the *NOP56* gene, additional information was collected retrospectively by contacting the recruiting clinician for each patient.

A total of 8763 genomes from patients recruited with hereditary ataxia (1257 patients from 1021 families) or controls (*n* = 7506) were analysed for the GGCCTG repeat in the *NOP56* gene. Controls were defined as probands without any neurological conditions over 40 years of age at the time of recruitment for the 100 000 Genomes Project.

Additional genetic screening of an in-house database consisting of 23 741 exomes from individuals with neurological diseases was also performed for *NOP56* expansions. Within the database, 1118 patients with ataxia were screened (of which 714 European, 147 South-East Asian, 90 African, 67 American and 8 East Asian based on genetic ancestry).

### *NOP56* genotyping from whole-genome sequencing

Short tandem repeat genotyping was performed using the ExpansionHunter software package version 3.2.2^23^ on all WGS/exome data. To estimate the size of the repeat in all individuals, we run ExpansionHunter against a custom json file (Supplementary material). The samples with detected expansions above 30 repeats were further visualized and manually inspected using GraphAlignmentViewer. The number of reads in the *NOP56* repeat region were calculated using BCFtools depth in the ataxia cohort of the exome database.

### Repeat-primed PCR

Triplet RP-PCR was performed to assess the presence of an expanded GGCCTG repeat as previously described by García-Murias *et al*.^6^ Reverse primers were used in equimolar concentrations attached to different points within the repeat expansion to produce multiple amplicons of incremental size. Fragment length analysis was performed on an ABI 3730xl Genetic Analyzer (Applied Biosystems), and data were analysed with the GeneMapper software (v. 4.0, Applied Biosystems). Expansions with characteristic decremental peaks indicating the presence of an expanded repeat pattern were identified as positives.

### Haplotype analysis

The ‘Aggv2’ file is an aggregated VCF file consisting WGS data from 78 195 individuals recruited for the 100 000 Genomes Project. These data have been phased using SHAPEIT4.2.2^24^ within the Genomics England Rare Diseases pipeline. The phased genotype calls were extracted from the Aggv2 VCF file for all five unrelated cases for a region of 1 Mb upstream and 1 Mb downstream of the *NOP56* repeat expansion using BCFtools version 1.12.^25^ To check for a shared haplotype between the five unrelated cases with the repeat expansion, the phased data were used to check for regions of highest sharing between the cases. A heatmap was plotted with regions of high sharedness between all subjects using Matplotlib^26^ (Supplementary Fig. 1). Using the *–indep-pairwise* function on PLINK^27^ with default parameters, the tagging single nucleotide variant (SNPs) within the shared haplotype block were identified using data from all participants in the Aggv2 VCF file (Supplementary Table 2).

### Ancestry

Participants were classified according to their genetic similarity into 21 reference groups curated using the UK Biobank as described in Privé *et al*.^28^ Specifically, the big_prodMat() function from the *bigsnpr* R package was used to project participant genotypes and reference group allele frequencies onto the top 16 linkage disequilibrium-scaled principal components (PCs) calculated using a selection of individuals from the UK Biobank and the 1000 Genomes Project across 5 816 590 loci aligned to the GRCh37 reference genome.^28^ Squared Euclidean distances between each participant and the 21 reference groups on the PC space were converted into approximate *F*_ST_,^28^ and participants were assigned to the genetically closest reference group when *F*_ST_ < 0.002 or unassigned if *F*_ST_ > 0.002 to all reference groups. For participant genotypes aligned to the GRCh38 reference genome, reference population allele frequencies and PC loadings were converted to GRCh38 coordinates using UCSC LiftOver prior to projection onto the PC space.

### Genome optical mapping

Briefly, blood was stored at −80°C and thawed in a water bath at 37°C. After a WBC quantitation was performed using HemoCue cuvette, the blood samples underwent cell lysis and proteinase K digestion. Samples were transferred on the Nanobind Disks, and gSNA was released upon the addition of isopropanol. After three wash steps, the disk was transferred to a fresh tube, and the gDNA was eluted. Limited shearing was applied to the ultra-high-molecular-weight gDNA, which was then mixed and equilibrated overnight at room temperature to facilitate DNA homogeneity. DNA labelling was processed using the Bionano Prep™ DLS DNA Labelling kit (Bionano Genomics) according to the kit protocol. In brief, the DL-Green fluorophore is attached to a specific sequence motif using the DLE-1 enzyme. After sequence-specific labelling with DLE-1, the labelled DNA is stained for backbone visualization. When imaged on the Saphyr, the labelled samples are visualized as green dots on blue lines. The labelled DNA was loaded on a Saphyr chip (Bionano Genomics) and run on a Saphyr instrument (Bionano Genomics).

### Statistical analyses

Data analysis was performed in R version 4.2.1. No statistical analyses were carried out in this study.

## Results

**Figure 1:**
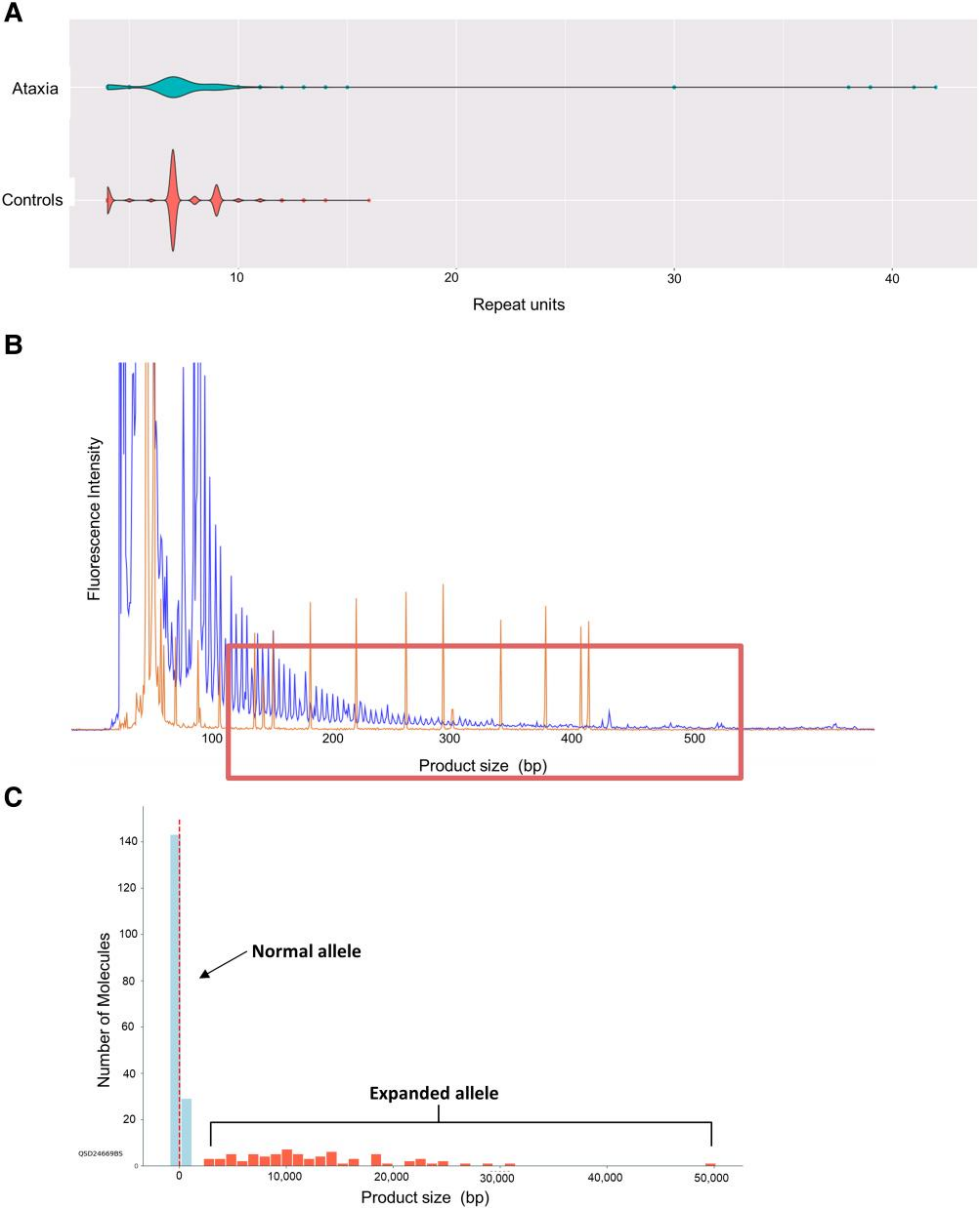
***NOP56* analysis in the 100 000 Genomes Project cohort, RP-PCR and optical genome mapping of the *NOP56* repeat expansion.** (**A**) Violin plots showing the distribution of the *NOP56* repeat size in the 100 000 Genomes Project cohort in hereditary ataxia cases in the top panel compared with controls in the bottom panel. (**B**) RP-PCR of sample II.1 from Family 2 carrying an expanded *NOP56* allele results in detrimental peaks indicated by the box. The *y*-axis shows the fluorescence intensity, while the *x*-axis shows the PCR product size in bp with each peak being a GGCCTG repeat. (**C**) Optical genome mapping results of the Patient II.1 from Family 2 confirm the presence of one normal and one expanded alleles. The genomic region around the GGCCTG repeat has been analysed (chr20: 2 651 701–2 659 012; markers 462–463, reference distance = 7990 bp). A peak of molecules representing the non-expanded allele and a variable number of larger molecules (expansion range 2696–30 515 bp, plus one outlier at 50 191 bp) are indicated by the arrows. *X*-axis is the distance between the DNA molecules and the reference inter-marker distance in base pairs. The number of repeats for an expanded allele is calculated as follows: [DNA molecules size (*x*-axis)−reference distance]/6.

Recently developed bioinformatic tools have shown that repeat expansions can be detected by WGS.^19^ The 100 000 Genomes Project was established in the UK to deliver WGS to patients with rare disease and cancer in the National Health Service, with over 75 000 patients with genetically undiagnosed rare disease recruited for the project. To analyse the GGCCTG repeat in the first intron of the *NOP56* gene in the UK population, we screened a total of 8763 WGS data from 1257 patients with hereditary ataxia and 7506 unrelated controls. We identified 7 patients from 5 families with expanded alleles over 30 copies of the GGCCTG repeat in the *NOP56* gene. By contrast, no expanded alleles (>30 GGCCTG repeats) were detected in the control group. The distribution of the non-expanded alleles ranged from 3 to 14 repeats, with the most common allele being 9 repeats (*n* = 5040; Fig. 1). No other expansions were identified in the in-house exome database comprising data from 23 741 individuals with neurological diseases, including 1118 with ataxia. The *NOP56* repeat region (chr20: 2 652 733–2 652 775) in the exome database was covered by an average of 30 reads across the ataxia cohort.

**Figure 2:**
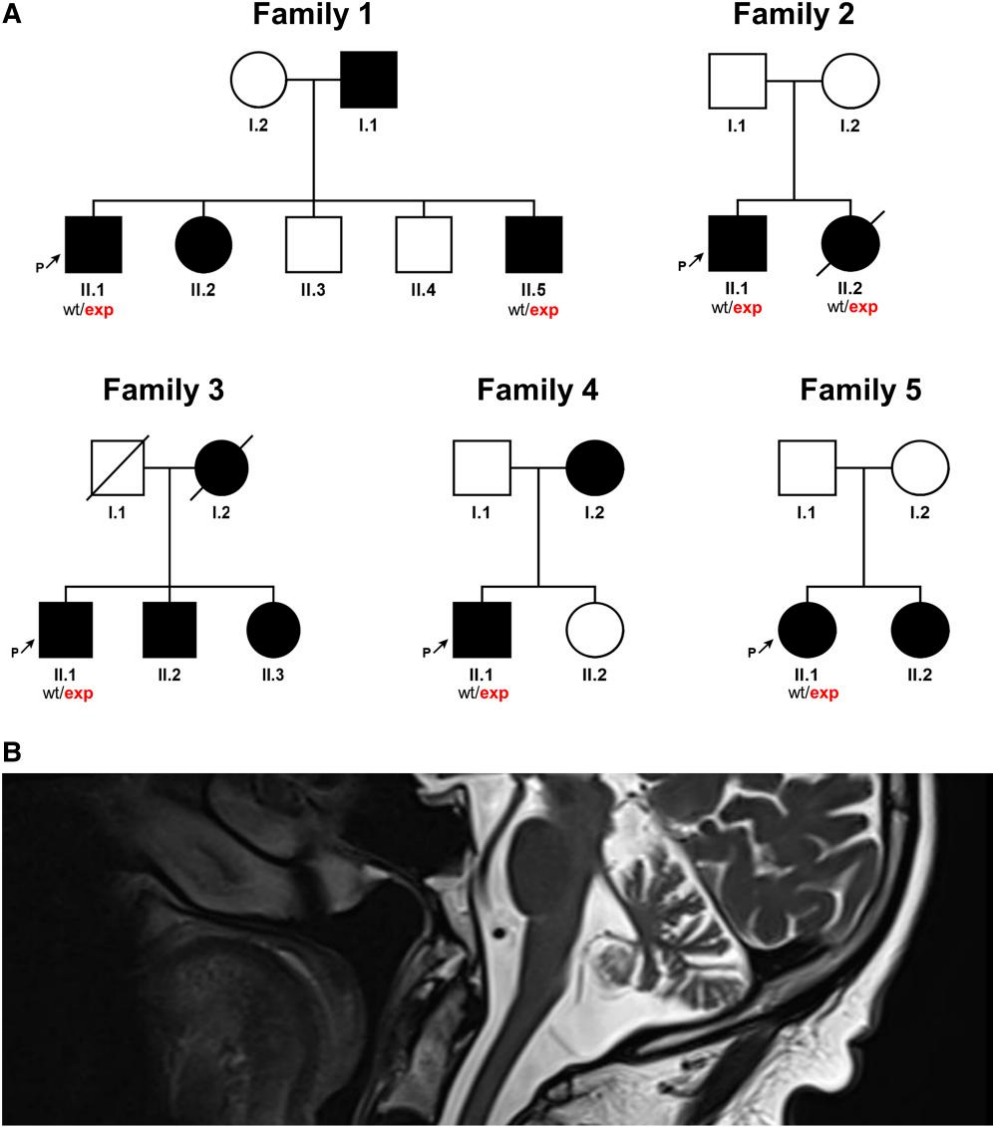
***NOP56* pedigrees and MRIs.** (A) Pedigrees of the five families described in the present study. Square, male; circle, female; black filled symbol, affected individual; white symbols, unaffected individuals; and diagonal line, deceased individual. (**B**) MRI brain of Patient II.1 (Family 2) demonstrating cerebellar atrophy.

All patient families demonstrated an autosomal-dominant pattern of inheritance (Fig. 2). These patients had also been screened for single-nucleotide and copy number variants in known ataxia genes (https://panelapp.genomicsengland.co.uk/panels/20/), and no other pathogenic/likely pathogenic variants had been identified.

The presence of the repeat expansion was confirmed by RP-PCR in all individuals from families 1–5 and showed the presence of the classical sawtooth pattern in all cases tested (Fig. 1). As both WGS and RP-PCR cannot accurately size large repeat expansions, we used optical genome mapping to size the expanded repeat on one sample. This confirmed the presence of a normal repeat and one expanded allele. The mean size of the expanded allele was 12 567 bp (median = 11 172). The data also suggest somatic instability of this repeat (Fig. 1).

The haplotype analysis performed on all unrelated individuals carrying the expanded repeat identified a shared haplotype of 72.2 kb that extends mainly in the distal direction (coordinates: chr20: 2 615 848–2 688 117, genome b38). This suggests a founder effect, and using a previously described method,^29^ we estimated this mutation to be 31.7 generations old (95% confidence interval: 16.9–60). The shared haplotype also comprises some common SNPs previously described in other populations,^4,6–8^ suggesting that there might be a much older common origin to this expanded repeat.

All seven patients with SCA36 were reported of White British descent; their ancestry was genetically confirmed to be White British (Table 1). The mean age of onset was 48.4 years (range 28–62 years); the disease duration ranged from 9 to 29 years, with the shorter durations due to death from other unrelated causes. All patients initially presented with gait ataxia, one patient had accompanying slurred speech and another had clumsiness. The progression of disease was slow, with all patients maintaining mobility after 9 years after onset, after which two patients died of other causes. The patient with the disease duration lasting 29 years continued to walk with support.

**Table 1:** Clinical features of UK SCA36 patients

	Family 1	Family 2	Family 3	Family 3	Family 4	Family 5	Family 5
PID	II.1	II.1	II.1	II.2	II.1	II.1	II.2
Year of birth	1957	1948	1944	1946	1963	1949	1950
Sex	M	M	M	M	M	F	F
Ethnicity	White British	White British	White British	White British	White British	White British	White British
Age at onset (years)	52	62	50s	50s	28	50	50
Age at examination (years)	61	70	70s	72	55	69	68
Disease duration	ND—died of heart failure, 9 years	12 years	20 years	ND—died from other causes	29 years	25 years	24 years
Initial symptom/s	Gait ataxia and slurred speech	Gait ataxia following myopathy during thyrotoxicosis	Balance disorder	Balance disorder	Gait ataxia	Gait ataxia	Gait ataxia and clumsiness
Progression	Slow	Slow	Slow	Slow	Slow	Slow	Slow
Years to being wheelchair bound	NA—still mobile	NA—still mobile	NA—still mobile	NA—died from other causes (mother wheelchair bound in her 70s)	NA—walks with wheeler	NA—walks with stick, unaided outdoors	ND—walks with frame/wheelchair
Hearing impairment (onset)		Yes—high frequency (50s)	ND	ND		No (bedside examination)	No (bedside examination)
Dysphagia		Yes	ND	Yes			
Dystonia			ND				
Dysautonomia		Yes (bladder)	ND				
Other	SARA score 6	Dyspnoea		AS, IBD sclerosing cholangitis			
Cognitive impairment		Prominent sub-cortical with very mild anterior cognitive compromise	ND	Very mild (MMSE 25/30) mother and sibs			
Ptosis		Yes	ND				
Cerebellar eye movement abnormality	Restricted—lateral gaze	Restricted—up gaze	ND	Nystagmus		Broken pursuit	Very slow saccades
Dysarthria	Yes	Yes	ND	Yes		Yes—very mild	Yes—mild
Tongue atrophy/fasciculation	ND		ND		ND		
Gait ataxia	Yes	Yes	Yes	Yes	Yes	Yes	Yes
Truncal ataxia		ND	ND			Yes—mild	Yes—moderate
Limb ataxia (dysmetria/dysdiadochokinesis)	Yes—mild	Yes	Yes	Yes	Yes	Yes—mild	Yes—moderate
Weakness	Yes (proximal)	Yes (hip flexors 4+)	ND				
Skeletal muscle atrophy		Yes (proximal)	ND				
Skeletal muscle fasciculation (UL/LL)			ND				
Spasticity			ND			Mild	Mild
DTRs: brisk (UL/LL)	UL/LL	UL/LL	ND		UL/LL	LL	UL/LL
Babinski	Normal	Normal	ND	Normal	Normal	Right extensor	Unreactive
Sensory loss		Reduced vibration to knees	ND				
Brain MRI	Cerebellar atrophy—mild	Cerebellar atrophy	ND	Cerebellar atrophy	Cerebellar atrophy—mild	Cerebellar atrophy—mild	ND
Other medical history	ND	Graves’ ophthalmoplegia following thyrotoxicosis age 60	ND	ND	ND	ND	ND

ND, not determined; NA, not applicable; UL, upper limb; LL, lower limb; AS, aortic stenosis; IBD, inflammatory bowel disease; SARA, scale for assessment rating of ataxia; DTR, deep tendon reflexes; MMSE, Mini-Mental State Examination.

Limb ataxia was present in all patients, while truncal ataxia was present in two. One patient lived abroad and could not be examined. Of the remaining six patients, at the time of examination at ages 55–72 years, five had dysarthria and two had dysphagia. Five patients had ocular involvement; two had restricted eye movements, and one patient each had broken pursuit, slow saccades and nystagmus. Hearing impairment was present in one patient, who presented with high tone frequency hearing impairment since his mid-50s. None of the patients had tongue fasciculation, but three patients were not specifically examined for this feature.

Upper limb tone and power were normal in all examined patients; while five of six patients had lower limb brisk reflexes, four also had upper limb brisk reflexes. In addition, one patient demonstrated lower limb spasticity, with unilateral extensor response. One patient demonstrated mildly reduced hip flexor strength with skeletal muscle atrophy and reduced vibration sense to the level of the knees. The same patient had bladder dysfunction. The patient had mild cognitive impairment, as did one other patient.

All patients who underwent brain MRIs showed cerebellar atrophy. The patient with ptosis also had Basedow's ocular myopathy. This patient also had a sister who was similarly affected and was also confirmed to carry the repeat expansion in SCA36.

## Discussion

We report for the first time a cohort of patients of White British descent with SCA36 and show that it accounts for 0.5% (5/1021) of hereditary ataxia cases that were unsolved by standard diagnostic testing in this population. Previous genetic screening of SCA36 in British patients with hereditary ataxia failed to identify *NOP56* expansions, probably due to the size of the cohort studied.^17^ The large-scale screening reported here using WGS data from 1257 patients with ataxia in the 10 000 Genomes Project allowed us to detect pathogenic repeat expansions in SCA36 patients and provide a genetic diagnosis in these families.

SCA36 was first reported in Western Japan and North Western Spain, with additional patients in the Han Chinese population and single cases of Vietnamese, French, Spanish and Portuguese descent. The mean age of onset in our UK cohort of SCA36 patients is in keeping with previous cohorts. The youngest symptomatic patient reported in the literature is 28 years old (Supplementary Table 1).^9^ The progression of disease in SCA36 varies, with multiple cohorts reporting slow progression. However in some patients, the years to wheelchair requirement was as short as 5 years,^9^ with several under 10 years.^9,30^

Similar to previous cohorts, our patients presented with gait ataxia. Dysarthria is a common feature (87%), as is eye involvement (65%) and hyper-reflexia (62%), consistent with our patients (Supplementary Fig. 2). Tongue fasciculation has been variable in previous cohorts (48% overall), particularly frequent in the initial reported cohorts in Japan and Spain.^6,14^ However, multiple subsequent reports have variably identified tongue fasciculations, similar to this cohort where none of our patients demonstrated this feature. Spasticity has been reported in a minority of patients (27%) and is present in two of our patients.

Hearing is variably affected in SCA36, being very frequent in some cohorts, with an overall occurrence of just under half of the patients previously reported. In the large cohort studied by García-Murias *et al*., hearing loss presented with initial symptoms, whereas in Japanese cohorts, hearing loss ranged from being not identified to 60%.^4,6,7^ Several studies of Han Chinese patients used formal testing to identify hearing loss, and Xie *et al.*^10^ noted that no patients in their cohort complained of hearing loss, but it was identified on audiometry.^8,11^ Only one of our patients was affected by hearing loss, but several were not formally examined.

Mild cognitive impairment can occur with SCA36, although Xie *et al.*^10^ recently described three patients with severe dementia. Our patients are in keeping with previous cohorts, which demonstrated a mild reduction in Mini-Mental State Examination scores.^4,6,8,10,31^ Impaired vibration sense present in one of our patients has been reported in 10% (15) of prior cohorts and to date is the only sensory modality impaired in SCA36.^4,8^ The same patient had bladder dysfunction, of which four prior patients have been described to have, potentially due to autonomic dysfunction.^6,10,31^ Similarly, dystonia has been reported in four patients with SCA36 and was not identified in our cohort.^10,13,30^

The majority of SCA36 cases demonstrate cerebellar atrophy on brain MRI, with a progressive involvement of the brainstem and cortical lobes in the advanced stages of the disease.^4,6,8,10,12,14,31^

In summary, we present evidence that SCA36 is a cause of hereditary ataxia in the British population, in patients presenting with slowly progressive cerebellar ataxia with high rates of dysarthria, eye involvement and hyper-reflexia and an absence of tongue fasciculations and hearing impairment, which were thought to be the cardinal features of SCA36.

## Supplementary Material

fcad244_Supplementary_Data

## Data Availability

Information about how to access the 100 kGP data by joining a Genomics England Clinical Interpretation Partnership is available online (www.genomicsengland.co.uk/join-a-gecip-domain). Exome data were accessed through our internal database.

## Appendix I

### Genomics England Research Consortium

Ambrose, J. C.; Arumugam, P.; Bevers, R.; Bleda, M.; Boardman-Pretty, F.; Boustred, C. R.; Brittain, H.; Brown, M. A.; Caulfield, M. J.; Chan, G. C.; Giess, A.; Griffin, J. N.; Hamblin, A.; Henderson, S.; Hubbard, T. J. P.; Jackson, R.; Jones, L. J.; Kasperaviciute, D.; Kayikci, M.; Kousathanas, A.; Lahnstein, L.; Lakey, A.; Leigh, S. E. A.; Leong, I. U. S.; Lopez, F. J.; Maleady-Crowe, F.; McEntagart, M.; Minneci, F.; Mitchell, J.; Moutsianas, L.; Mueller, M.; Murugaesu, N.; Need, A. C.; O’Donovan, P.; Odhams, C. A.; Patch, C.; Perez-Gil, D.; Pereira, M. B.; Pullinger, J.; Rahim, T.; Rendon, A.; Rogers, T.; Savage, K.; Sawant, K.; Scott, R. H.; Siddiq, A.; Sieghart, A.; Smith, S. C.; Sosinsky, A.; Stuckey, A.; Tanguy, M.; Taylor Tavares, A. L.; Thomas, E. R. A.; Thompson, S. R.; Tucci, A.; Welland, M. J.; Williams, E.; Witkowska, K.; Wood, S. M. and Zarowiecki, M.
