# Supplementary material for: Repeat expansions in NOP56 are a cause of spinocerebellar ataxia Type 36 in the British population
Source: Brain Commun. 2023 Sep 14;5(5):fcad244. doi: 10.1093/braincomms/fcad244 (PMC10558097; doi:10.1093/braincomms/fcad244)
Supplement: fcad244_Supplementary_Data [file fcad244_supplementary_data.zip › Supplementary Material SCA36.docx]

NOP56 genotyping from whole genome sequencing

ExpansionHunter software package version 3.2.2 custom made json file

[

{

“LocusId”: “NOP56”,

“LocusStructure”: “(GGCCTG)*(CGCCTG)*”,

“ReferenceRegion”:[

“chr20:2652733-2652757”,

“chr20:2652757-2652775”

],

“VariantId”: [

“NOP56”,

“NOP_CGCCTG”

],

“VarianType”: [

“Repeat”,

“Repeat”,

]

}

]
