# Supplementary material for: Repeat expansions in NOP56 are a cause of spinocerebellar ataxia Type 36 in the British population
Source: Brain Commun. 2023 Sep 14;5(5):fcad244. doi: 10.1093/braincomms/fcad244 (PMC10558097; doi:10.1093/braincomms/fcad244)
Supplement: fcad244_Supplementary_Data [file fcad244_supplementary_data.zip › Supplementary_figure_and_table_legends.docx]

**Supplementary figure and table legends**

**Supplementary Figure 1: Haplotype analysis of the *NOP56* repeat region in cases and controls showing a 72.2kb shared region on chromosome 20.**

The heatmap comprises a shared haplotype between the 5 unrelated cases (blue represents reference allele; yellow represents the alternate allele) that spans chr20:2615848-2688117 (genome build 38), with each vertical line representing a shared SNP. The red line represents the *NOP56* repeat expansion at chr20:2655000.

**Supplementary Figure 2: Clinical Features of SCA36: British cohort compared to previous cases.** Blue = features is present, Orange = feature is absent, Grey = undetermined. Comparison calculated from published cases data summarised in Supplementary Table 1.

**Supplementary Table 1: Literature review of SCA36 published cases.**

**Supplementary Table 2: List of 9 tagging SNPs in the 72.2Kb-long shared haplotype block between 5 unrelated SCA36 cases.**
