# Supplementary figures and images for: Repeat expansions in NOP56 are a cause of spinocerebellar ataxia Type 36 in the British population
Source: Brain Commun. 2023 Sep 14;5(5):fcad244. doi: 10.1093/braincomms/fcad244 (PMC10558097; doi:10.1093/braincomms/fcad244)

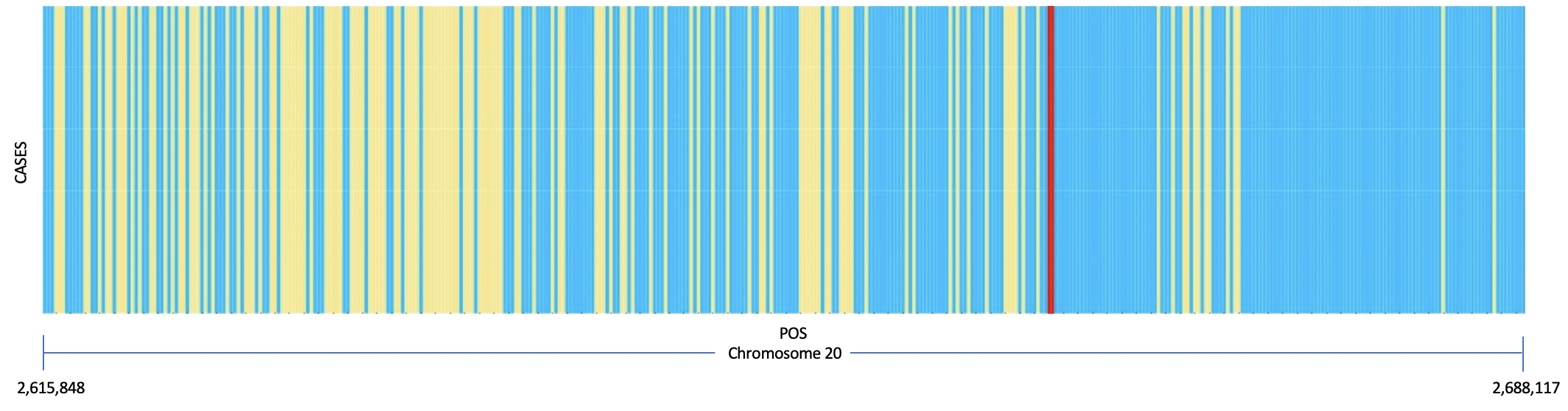

Supplement: fcad244_Supplementary_Data [file fcad244_supplementary_data.zip › Supplementary Figure 1.png]

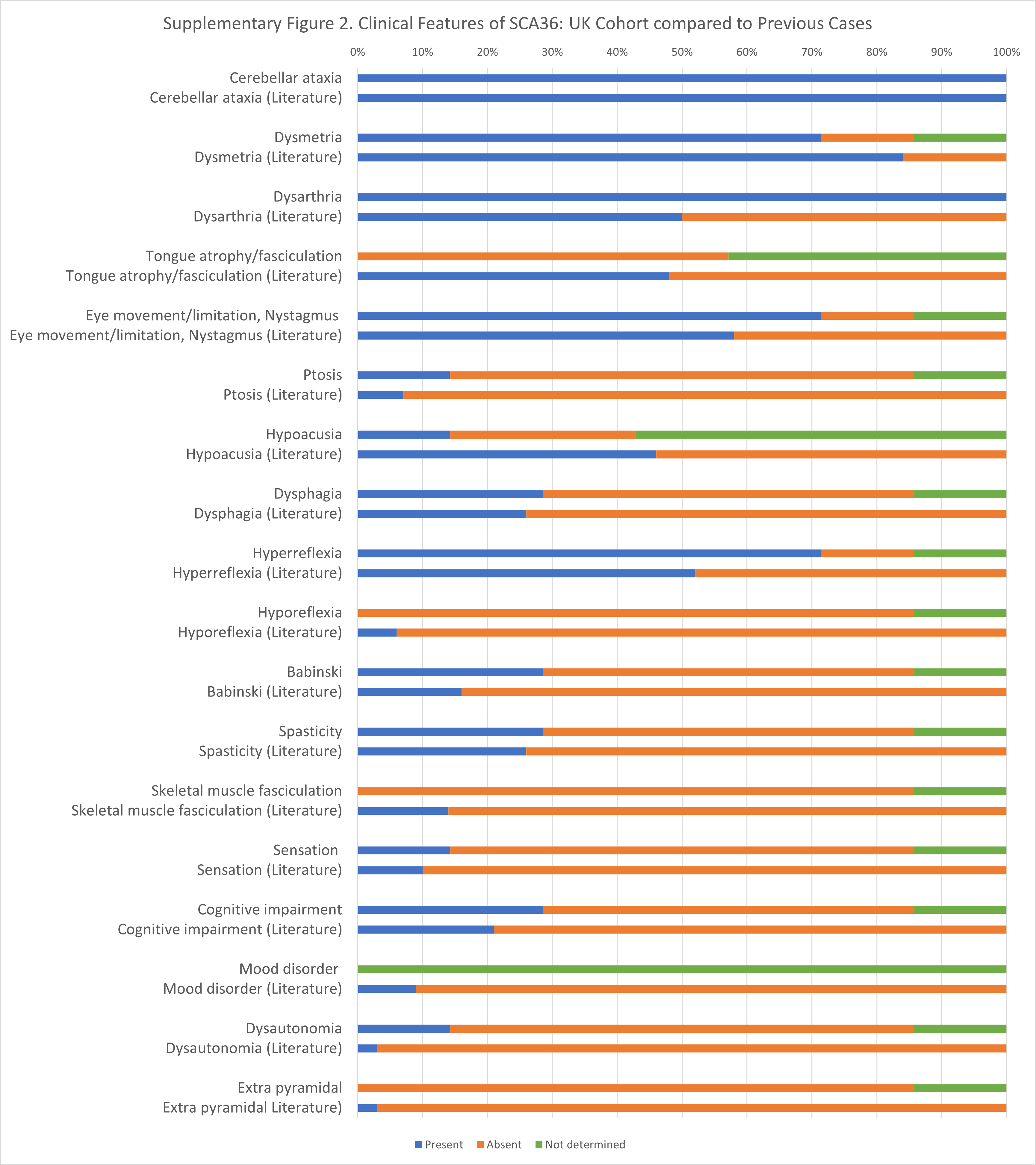

Supplement: fcad244_Supplementary_Data [file fcad244_supplementary_data.zip › Supplementary Figure 2.png]
